# Prognosis Risk of Urosepsis in Critical Care Medicine: A Prospective Observational Study

**DOI:** 10.1155/2016/9028924

**Published:** 2016-02-03

**Authors:** Xin-Hua Qiang, Tie-Ou Yu, Yi-Nan Li, Li-Xin Zhou

**Affiliations:** Department of Critical Care Medicine, The First People's Hospital of Foshan, Foshan, Guangdong 528000, China

## Abstract

This study aimed to investigate the clinical features of urosepsis and to raise awareness of this problem. Of the 112 sepsis patients enrolled, 36 were identified as having urosepsis. The bacteria involved in the infection leading to urosepsis included* Escherichia coli*,* Proteus* species,* Enterococcus* species,* Klebsiella* species, other Gram-positive cocci, and* Pseudomonas aeruginosa*. Although the organ/system dysfunction appeared earlier in the urosepsis patients than in the other sepsis patients (4.7 ± 2.4 versus 7.2 ± 4.5 hours, *P* < 0.001), the urosepsis patients presented with a better prognosis and lower 28-day mortality rate than the others (6% versus 37%). In the multivariate analysis, the type of sepsis (urosepsis, OR = 0.019, 95% CI = 0.001, 0.335, *P* = 0.007) and SOFA score (OR = 1.896, 95% CI = 1.012, 3.554, *P* = 0.046) remained significantly associated with the survival. The time of admission to the intensive care unit of 17 patients transferred from the Department of Urinary Surgery was significantly prolonged compared with those transferred from other departments (11.6 ± 7.3 versus 7.2 ± 4.9 hours, *P* < 0.05). In conclusion, urosepsis suggested a better prognosis, but attention needs to be paid in clinical practice, especially in urinary surgery.

## 1. Introduction

Urosepsis is sepsis that derives from a urogenital tract infection and is a common problem that has been documented for a long time [1]. It was not defined until 2010 by the European Association of Urology (EAU) [2, 3]. In 20%–30% of sepsis patients, the infection originates from the urinary tract, and urosepsis often develops from urinary tract infections (UTIs) acquired in a community or hospital [4]. The globally accepted mortality rate of severe sepsis is 20%–42%. About 50% of severe sepsis originates from pneumonia, with 24% from intraperitoneal infection and 5%–7% from UTI [5]. Similar to sepsis induced by other types of infections, the severity of urosepsis is also closely related to a patient's immune function. A 10-year study of urosepsis shock [6], that is, low blood pressure and decreased oxygen flow due to severe sepsis, reported that 78% of 59 patients (54% females) presented with urinary tract obstruction, 22% presented with obvious urination disorder, and 17% presented after receiving surgical intervention. Also, the pyelonephritis induced by obstructive diseases may be caused by urinary stones (65%), tumors (21%), gestation (5%), urinary tract anomalies (5%), and surgery (4%) [7]. In summary, the following are high-risk factors for urosepsis [8]: old age, female gender, diabetes, immunosuppression (organ transplantation), use of chemotherapy or steroids, AIDS, chronic renal failure, anemia, diameter of stone >2.5 cm, and extremely long operation time.

The objectives of this study are to investigate the clinical features of urosepsis and to raise awareness of this problem so that it receives the required attention from urologists and intensivists.

## 2. Materials and Methods

### 2.1. Subjects

This was a prospective cohort study. It included 112 subjects with severe sepsis between June 2010 and August 2013, according to the American College of Chest Physicians/Society of Critical Care Medicine guidelines. The inclusion criteria for this study were patients of 18–80 years of age and with an accurate diagnosis of severe sepsis. The exclusion criteria were as follows: (1) malignant tumor, (2) chronic renal failure, and (3) cerebral hemorrhage or cerebral infarction. Of the 112 patients, 36 were diagnosed with urosepsis, called the urosepsis group, and the rest with other types of sepsis were considered as the control group. All subjects included in the study provided written informed consent. The study protocol was approved by the Ethics Committee of The First People's Hospital of Foshan, China.

### 2.2. Demographic Data Collection

Demographic information, including patients' age and gender and disease-related characteristics such as time when the organ/system dysfunction occurred and time when intensive medicine doctors were asked for assistant diagnosis and clinical outcomes, was collected for both the groups. Acute Physiology and Chronic Health Evaluation (APACHE) II score, Sequential Organ Failure Assessment (SOFA) score, HLA-DR, concomitant diseases, and standard biochemical tests were also assessed.

### 2.3. Statistical Methods

The SPSS 17.0 software (SPSS Inc., IL, USA) was used for analyzing data. Measured data were expressed as mean ± standard deviation ($\bar{x}\pm s$) and were compared using the *t*-test. Count data were compared using *χ* ^2^ test. *P* < 0.05 was considered as significant. Multiple logistic analyses were conducted to evaluate the risk factor for urosepsis.

## 3. Results

### 3.1. Demographics and Characteristics

Both the urosepsis and control groups showed different background demographics such as age and gender. APACHE II scores reflecting the severity of the cases, the SOFA scores evaluating organ/system dysfunction, and the HLA-DR indicating the immunological status of sepsis patients were not significantly different between the two groups (Table 1).

### 3.2. Disease Severity upon Entering ICU

The comparison of disease severity between the two groups upon entering the ICU within 24 hours of hospital admission is shown in Table 1. The organ/system dysfunction in the urosepsis group was found to occur earlier than in the control group (4.7 ± 2.4 versus 7.2 ± 4.5 hours, *P* < 0.001). Further, the white blood cell counts and platelets significantly declined and procalcitonin (PCT) as an inflammation indicator significantly increased (all in *P* < 0.001) in the urosepsis group, but time to enter the ICU, bilirubin, mean arterial pressure (MAP), creatinine, and oxygenation index were not significantly different from those of the control group.

During intra-ICU treatment, the two groups were not significantly different in the use of vasoactive drugs for maintaining blood pressure, continuous renal replacement therapy (CRRT), or liver assist devices (Table 1). The proportion of mechanical ventilation increased in the control group, but, in the urosepsis group, the positive rate of blood culture was extremely high whether the urosepsis patients entered the ICU after operation or via other routes. So, the study concluded that the urosepsis group with a shorter ICU stay, favorable prognosis, and a lower 28-day mortality rate (6% versus 37%, *P* < 0.001) was better than the control group.

### 3.3. Multivariate Analysis

To find out the risk factors associated with mortality in the sepsis patients, clinical and investigative variables such as type of sepsis patients, gender, age, combination disease, positive blood cultures, APACHE II score, SOFA score, and time before being sent to ICU were first evaluated by the univariate analysis. The factors that were significant in the univariate analysis were then subjected to multivariate logistic regression analysis. In the multivariate analysis, six variables with statistical differences on the univariate analysis were included: type of sepsis, age, combination disease, APACHE II score, SOFA score, and time before being sent to ICU (Table 2). The type of sepsis (urosepsis) and SOFA score remained significantly associated with survival (OR = 0.019, 95% CI = 0.001, 0.335, *P* = 0.007 and OR = 1.896, 95% CI = 1.012, 3.554, *P* = 0.046, resp.), but the other variables showed a tendency to be associated with the infection.

### 3.4. Subgroup Analysis of Urosepsis

For the subgroup analysis of urosepsis, the 17 cases transferred to the Department of Critical Care Medicine from the Department of Urinary Surgery were compared with those transferred from other departments (*n* = 19, control-urosepsis group), including the nephrology, emergency, or geriatric departments. In the 17 cases transferred, the intensivists spent significantly longer time in assisting diagnosis and treatment before transfer compared with the control-urosepsis group (11.6 ± 7.3 versus 7.2 ± 4.9 hours, *P* < 0.05). Other indicators such as APACHE II scores, SOFA scores, mechanical ventilation use, CRRT, vasoactive drug use, white blood cell count, platelet count, MAP, creatinine level, bilirubin level, positive blood culture rate, or mortality rate were not significantly different from the control-urosepsis group (Table 3).

## 4. Discussion

The aim of this study was to describe the clinical features of severe urosepsis patients to raise awareness of this form of sepsis. Despite the fact that urosepsis is associated with a good prognosis and low mortality, it must be remembered that the basis of a successful sepsis therapy is a short time to treatment [14].

This study showed significantly different demographics between the urosepsis group and the control group. This was probably because a proportion of the patients in the urosepsis group had received surgical treatment and was relatively young, and thus the number and proportion of basic diseases were smaller in this group than in the control group. The difference in gender distributions was due to the fact that females were more prone to urinary infections [8]. In the present study, the proportion of females (53%) was basically similar to previous studies, but the proportion of postoperative patients was obviously increased, probably because The First People's Hospital of Foshan is the only Grade III Class A hospital in Foshan city, and thus all difficult surgeries throughout Foshan are transferred and performed in the Department of Urinary Surgery of this hospital.

Multivariate logistic regression analysis was used when the variables were significant in the univariate analysis. In the present study, six variables were included in the multivariate analysis, and the type of sepsis (urosepsis) and SOFA score remained significantly associated with the survival, while age, combination disease, APACHE II score, and time before being sent to ICU showed a tendency to be associated with the survival.

The bacteria responsible for urosepsis include* Escherichia coli*,* Proteus* species,* Enterococcus* species,* Klebsiella* species, other Gram-positive (G+) cocci, and* Pseudomonas aeruginosa*. For patients with obvious immune dysfunction, monilia (*Candida albicans*) yeast infection or* Pseudomonas* may appear in the bacterial culture. The classifications of causes in this study were similar to those reported previously, but the bacterial compositions were obviously different, as* E*.* coli* accounted for 58% (*n* = 21),* Proteus* species bacillus vulgaris accounted for 8% (*n* = 3),* Enterococcus* species and* Klebsiella* species accounted for 11% (*n* = 4), other G+ cocci accounted for only 6% (*n* = 2), and 17% (*n* = 6) had no culture results. A comparison of the pathogenic bacterium involved in urosepsis found in this study and published in previous studies is presented in Table 4, although most of the previous studies did not distinguish between patients who developed urosepsis and those who had UTI with the risk of developing urosepsis [9–13]. Table 4 shows that different studies had different rates of infection with different bacterial species. The differences may be attributed to population differences and geographical differences as well as the fact that most cases in the current study were first-onset. Understanding the bacteria involved in the development of urosepsis is an important part of developing successful antibiotic treatment therapies to either avoid the development of urosepsis from UTI or aid the fast treatment once urosepsis has developed [12]. As all these studies suggest that urosepsis is most commonly caused by* E*.* coli* (in contrast to the other sepsis patients in the present study), this should be considered by the use of suitable antibiotics in high-risk patients [12].

The diagnosis tools for urosepsis are not complex [2, 3]; however, urosepsis occurs and develops rapidly. The results of this study show that, upon entering the ICU, the white blood cell counts obviously declined among 64% of patients (*n* = 23) and the platelet counts dropped in 53% of patients. However, PCT rose significantly, and the test results in 33% of patients (*n* = 12) exceeded the 200 ng/mL threshold, much higher than the values needed to suggest urosepsis [13], probably because the patients in the urosepsis group were younger and presented with fewer basic diseases compared with the control group. However, the two groups were not significantly different in the severity of the disease, incidence of organ/system dysfunction, SOFA scores, or HLA-DR. The organ/system dysfunction in the urosepsis group occurred obviously earlier than in the control group. Since the urinary blood supply was rich and the blood culture positive rates were obviously high, the urosepsis group was considered to suffer from bacteremia and/or blood poisoning immediately after the onset, thus rapidly leading to organ/system dysfunction.

According to the EAU* Guidelines for Diagnosis and Treatment of Urinary Calculi* 2006, blood poisoning is induced by overly high pressure in the renal collecting system, which is due to intraoperative operational problems. It should be noted that, with calculus complicated by infection, intraoperatively rapid perfusion and washing will increase intrarenal pressure and probably allow bacteria or toxins to enter blood and induce bacteremia or toxemia [15]. The appropriate measures include the preventive use of antibiotics before operation, smoothing the outflow of perfusate during operation, and reduction of hypoperfusion pressure. Urosepsis is the most severe complication for percutaneous nephrolithotomy, and the incidence rates of relevant complications are as follows: bacteremia (23%), endotoxemia (34%), fever (25%), and septic shock (0.3%–2.5%) [16]. It should be noted that, among hospital-acquired UTIs treated by urinary surgery, the mean incidence rate of urosepsis was 12%, but, in other fields, the incidence rates of severe sepsis and septic shock were 2% and 0.3%, respectively [17]. The statistically analyzed mortality rate of urosepsis was 25%–60%, but, in reality, compared with septic shock induced by infections in other organs/systems, the mortality rate of UTI-induced septic shock was obviously lower; despite the lack of definite evidence, such phenomenon is very likely correlated with surgical drainage [18]. In the subgroup analysis of urosepsis, among the 17 cases transferred from the Department of Urinary Surgery to the Department of Critical Care Medicine, the intensive medicine doctors spent significantly longer time in assistant diagnosis and treatment than in the control group, which was probably related to inadequate postoperative observation and insufficient attention.

## 5. Conclusions

Urosepsis is generally reported to have low mortality rates and favorable outcomes compared with sepsis induced in other organ/system or tissues. However, enough attention needs to be paid to it, especially in the surgery departments.

## Figures and Tables

**Table 1:** Demographic data and clinical features of the urosepsis and the control groups.

	Urosepsis (*n* = 36)	Control (*n* = 76)	*χ* ^2^/*t*	*P* value
Age (years)	43.6 ± 12.5	57.3 ± 19.3	−3.886	<0.001
Gender (male/female, %)	17/19 (47%/53%)	45/31 (59%/41%)	1.421	0.233
APACHE II score	20.3 ± 5.6	21.8 ± 6.1	−1.247	0.215
SOFA score	5.8 ± 1.9	5.1 ± 2.2	1.640	0.104
HLA-DR (%)	56.47 ± 21.63	49.85 ± 24.47	1.386	0.168
Combination diseases	1.02 ± 0.84	2.36 ± 1.44	−5.174	<0.001
Time before being sent to ICU (hours)	9.2 ± 6.6	7.2 ± 4.9	1.798	0.075
Organ dysfunction time (hours)	4.7 ± 2.4	7.2 ± 4.5	−3.124	0.002
WBC count (×10^9^/L)	6.3 ± 5.5	14.7 ± 4.8	−8.249	<0.001
PLT count (×10^9^/L)	83.2 ± 34.6	138.9 ± 45.1	−6.548	<0.001
Serum creatinine (*μ*mol/L)	125.9 ± 49.8	121.2 ± 56.4	0.427	0.670
Bilirubin (*μ*mol/L)	15.9 ± 3.8	16.4 ± 3.2	−0.726	0.469
Mean arterial pressure (mmHg)	92.8 ± 19.2	99.3 ± 17.7	−2.038	0.053
Oxygenation index	214.1 ± 45.3	208.3 ± 56.5	0.539	0.591
PCT (ng/mL)	129.3 ± 40.8	41.2 ± 38.4	11.114	<0.001
ICU stay (days)	11.6 ± 5.5	14.8 ± 7.1	−2.384	0.019
Total hospitalization (days)	16.2 ± 6.9	18.1 ± 9.2	−1.100	0.274
Mechanical ventilation	20 (56%)	53 (70%)	2.165	0.141
Vasoactive drugs use	17 (47%)	29 (38%)	0.829	0.362
CRRT	10 (28%)	21 (28%)	0.000	0.987
Extracorporeal liver assist device (artificial liver)	2 (6%)	5 (7%)	0.044	0.834
Positive blood cultures	27 (75%)	23 (30%)	19.783	<0.001
28-day mortality	2 (6%)	28 (37%)	12.193	<0.001
90-day mortality	2 (6%)	32 (42%)	15.436	<0.001

APACHE, Acute Physiology and Chronic Health Evaluation; SOFA, Sequential Organ Failure Assessment; ICU, intensive care unit; WBC, white blood cell; PLT, platelet; PCT, procalcitonin; CRRT, continuous renal replacement therapy.

**Table 2:** Univariate and multivariate logistic analysis for evaluating the prognosis for sepsis.

	Surviving	Dead	Univariate analysis	Multivariate analysis		
	*n* = 82	*n* = 30	(*P* value)	OR	95% CI	*P* value
Type of sepsis (percentage of urosepsis, %)	41.5% (34/82)	6.7% (2/30)	<0.001	0.019	0.001, 0.335	0.007
Age (year)	46.66 ± 15.58	69.60 ± 15.33	<0.001	1.024	0.959, 1.019	0.483
Gender (male/female)	45/37	17/13	0.870			
Combination disease (*n*)	1.41 ± 1.45	3.33 ± 0.76	<0.001	1.298	0.485, 3.479	0.604
Positive blood cultures (%)	40.2%	56.7%	0.122			
APACHE II score	19.21 ± 5.12	27.47 ± 4.01	<0.001	1.131	0.871, 1.469	0.354
SOFA score	4.61 ± 1.73	7.30 ± 1.97	<0.001	1.896	1.012, 3.554	0.046
Time before being sent to ICU (hours)	7.04 ± 4.82	10.13 ± 5.65	<0.001	0.963	0.823, 1.126	0.634

**Table 3:** Subgroup analysis of urosepsis patients.

	Transferred from the department of urinary surgery (*n* = 17)	Transferred from other departments (*n* = 19)	*χ* ^2^/*t*	*P* value
Time before being sent to ICU (hours)	11.6 ± 7.3	7.2 ± 4.9	2.239	0.032
Positive blood culture rate	14 (82%)	13 (68%)	0.929	0.335
Percentage with combination diseases	5 (29%)	11 (58%)	2.948	0.086
SOFA score	5.7 ± 2.1	6.0 ± 1.8	−0.462	0.647
APACHE II score	18.8 ± 6.2	22.4 ± 5.6	−1.831	0.076
Mechanical ventilation	9 (53%)	11 (58%)	0.089	0.765
CRRT	4 (24%)	6 (31%)	0.290	0.590
Vasoactive drugs use	8 (47%)	9 (47%)	<0.001	0.985
WBC count (×10^9^/L)	5.9 ± 5.1	6.4 ± 5.7	−0.276	0.784
PLT (×10^9^/L)	81.0 ± 35.6	85.7 ± 32.3	−0.415	0.680
MAP (mmHg)	89.4 ± 20.5	93.2 ± 18.8	−0.580	0.566
Serum creatinine (*μ*mol/L)	123.6 ± 50.9	126.4 ± 48.6	−0.169	0.867
Bilirubin (*μ*mol/L)	16.8 ± 4.2	14.5 ± 3.7	1.747	0.090
28-day mortality	1 (6%)	1 (5%)	0.007	0.935
90-day mortality	1 (6%)	1 (5%)	0.007	0.935

APACHE, Acute Physiology and Chronic Health Evaluation; SOFA, Sequential Organ Failure Assessment; ICU, intensive care unit; WBC, white blood cell; PLT, platelet; MAP, mean arterial pressure; CRRT, continuous renal replacement therapy.

**Table 4:** Comparison of pathogenic bacterium in this study and others.

	Current study		Johansen et al. 2006 [9]	Bishara et al. 1997 [10]	van Nieuwkoop et al. 2010 [11]	DasGupta et al. 2009 [12]	Sugimoto et al. 2013 [13]
	Urosepsis *n* = 36	Sepsis^*∗*^ *n* = 76	Urosepsis	Urosepsis/UTI	Urosepsis/UTI	UTI	Urosepsis
*Escherichia coli*	21 (58%)	10 (13.2%)	50%	52%	79%	45%	51%
*Proteus *species	3 (8%)	6 (7.9%)	15%	9%	4%	10%	3%
*Enterococcus *species	4 (11%)	20 (26.3%)	15%	14%	7%	25%	27%
Other G+ cocci	2 (6%)	12 (15.8%)	15%	—	2%	6%	11%
*Pseudomonas aeruginosa*	0	24 (31.6%)	5%	8%	2%	8%	5%
No culture results	6 (17%)	23 (30.3%)	—	—	—	—	—

^*∗*^There were multiple infections in some patients, so the total percentage is more than 100%.
